# Different evolutionary trends of swine H1N2 influenza viruses in Italy compared to European viruses

**DOI:** 10.1186/1297-9716-44-112

**Published:** 2013-12-01

**Authors:** Ana Moreno, Elena Gabanelli, Enrica Sozzi, Davide Lelli, Chiara Chiapponi, Massimo Ciccozzi, Gianguglielmo Zehender, Paolo Cordioli

**Affiliations:** 1 Department of Virology, Istituto Zooprofilattico Sperimentale della Lombardia ed Emilia Romagna, Via Bianchi, 9, 25124 Brescia, Italy; 2 Laboratory of Infectious Diseases and Tropical Medicine, University of Milan, Ospedale Luigi Sacco Azienda Ospedaliera Polo Universitario, Via G.B. Grassi, 74, 20157 Milan, Italy; 3 Diagnostic Laboratory, Istituto Zooprofilattico Sperimentale della Lombardia ed Emilia Romagna, Via dei Mercati, 13A, 43100 Parma, Italy; 4 Department of Infectious, Parasitic, and Immunomediated Disease, National Institute of Health, V.le Regina Elena, 299, 00161 Rome, Italy

## Abstract

European H1N2 swine influenza viruses (EU H1N2SIVs) arose from multiple reassortment events among human H1N1, human H3N2, and avian influenza viruses. We investigated the evolutionary dynamics of 53 Italian H1N2 strains by comparing them with EU H1N2 SIVs. Hemagglutinin (HA) phylogeny revealed Italian strains fell into four groups: Group A and B (41 strains) had a human H1 similar to EU H1N2SIVs, which probably originated in 1986. However Group B (38 strains) formed a subgroup that had a two-amino acid deletion at positions 146/147 in HA. Group C (11 strains) contained an avian H1 that probably originated in 1996, and Group D (1 strain) had an H1 characteristic of the 2009 pandemic strain. Neuraminidase (NA) phylogeny suggested a series of genomic reassortments had occurred. Group A had an N2 that originated from human H3N2 in the late 1970s. Group B had different human N2 that most likely arose from a reassortment with the more recent human H3N2 virus, which probably occurred in 2000. Group C had an avian-like H1 combined with an N2 gene from one of EU H1N2SIVs, EU H3N2SIVs or Human H3N2. Group D was part of the EU H3N2SIVs clade. Although selection pressure for HA and NA was low, several positively selected sites were identified in both proteins, some of which were antigenic, suggesting selection influenced the evolution of SIV. The data highlight different evolutionary trends between European viruses and currently circulating Italian B strains and show the establishment of reassortant strains involving human viruses in Italian pigs.

## Introduction

Influenza A viruses of subtypes H1N1, H3N2 and H1N2 have been reported in pig populations around the world. Unlike human influenza, the origin and nature of swine influenza viruses (SIV) differ between continents. Indeed, two lineages of SIV that are characterized by distinct genomic evolutions are recognized: the Eurasian lineage that circulates in Europe and Asia, and the American lineage that is predominant in America but is also present in Asia. In recent years, the epidemiology of European SIV has changed considerably; the prevalent H1N1 viruses in European countries are antigenically distinct from the classical H1N1 strains and apparently stem from the introduction of an avian virus *in toto*[1]. These avian-like viruses, which appeared in European mainland pigs in 1979, seem to have a selective advantage over classical viruses, because in Europe they have replaced the classical SIV [2]. The H3N2 viruses that have been present in Europe since 1984 resulted from a genomic reassortment between human-like swine H3N2 viruses and avian-like swine H1N1 viruses. These viruses are characterized by the presence of human hemagglutinin (HA) and neuraminidase (NA), whereas the internal genes are all avian in origin [2,3]. The latest detected subtype is H1N2, which was introduced into the swine population in Europe at two different times. H1N2 SIV were first isolated in France in 1987 and 1988, and arose from a genomic reassortment between avian-like H1N1 SIV and human H3N2 viruses [4]. However, these strains did not spread beyond their farms of origin. The H1N2SIV currently circulating in Europe are derived from those isolated in Great Britain in 1994 [5]. They contain an HA gene closely related to that of human H1N1 viruses, an NA gene derived from human H3N2 viruses, and internal genes of avian origin [6]. The H1N2 SIV quickly spread to pigs in the rest of Europe [7,8] and became endemic. In Italy, the continuous circulation of the H1N1, H1N2, and H3N2 SIV subtypes in pig populations has been reported [3,7,9]. Swine monitoring programs at the Istituto Zooprofilattico Sperimentale della Lombardia e dell'Emilia Romagna (IZSLER) have been in place since the 1990s and are based on genome detection, virus isolation and sequencing of all respiratory forms. Surveillance performed from 1998 to 2012 revealed there was a continuous circulation of H1N1, H3N2 and H1N2 viruses and isolation of the H1N1 pdm viruses in pigs starting in 2009. The most frequent subtype was the avian-like H1N1, followed by H3N2. The H1N2 subtype was first isolated in Italy in 1998 but the number of H1N2 isolations has increased over the last five years, and in 2009–2010 it was one of the most frequently detected subtypes. During this time it represented 37% of all isolations, compared to 35% for H1N1 and 28% for H3N2 [10].

Genomic reassortment between different influenza subtypes is considered to be one of the evolutionary mechanisms that generate novel virus strains that have a pandemic potential for human populations. Swine were suspected to be a reassortment location for human and avian viruses, and to be a reservoir for viral variants that have the potential to produce pandemic human strains [11]. Recently, the global and local molecular clock concept in a maximum likelihood framework was used to confirm that human NA, HA and other internal protein genes had been introduced into European swine H1N2 lineages in the 1970s, early 1980s and late 1980s, respectively, through different reassortments [12]. However, only H1N2SIV isolated in Europe up until 2001 were included in this study, so it lacked data on the evolutionary dynamics of H1N2 SIV that began circulating in more recent years. In order to better understand the epidemiology and molecular evolution of H1N2 SIV in Italy, we characterized the HA and NA genes of 53 Italian H1N2 strains isolated from 1998 to 2012. Phylogenetic analysis was carried out by comparing them with the HA and NA sequences of influenza viruses (IV) isolated from swine, humans and avian species available in Influenza Virus Resource at the National Center for Biotechnology information. Furthermore, we reconstructed the evolutionary dynamics of influenza A viruses of human and swine origin using the Bayesian Markov Chain Monte Carlo method. From this, we estimated both the rates of nucleotide substitution and Time to the Most Recent Common Ancestor (TMRCA) for the HA and NA genes. The last part of the study investigated site by site positive selection pressures by estimating the rates of dN and dS substitutions in the HA and NA genes in order to identify the main mutations that allowed viral immune-escape. These findings provide valuable information about the evolutionary processes of influenza A viruses of human and swine origin, including the impact the reassortment events have had on the evolutionary history of the H1N2 viruses. Moreover, these data highlight the different evolutionary trends of Italian strains compared to circulating European viruses, showing that reassorted strains, which involved human viruses, are now established in pig populations in Italy.

## Materials and methods

### Virus isolation and subtype determination

Nasal swabs or lungs were collected from pigs that showed clinical signs and/or had lesions related to swine influenza. The samples were tested for influenza A using real-time RT-PCR as previously described by Spackman et al. [13]. For virus isolation, positive samples were inoculated into Madin-Darby canine kidney (MDCK) and CACO-2 cells and propagated through the allantoic sac route of 9–11 day old SPF chicken embryonated eggs (CEE). Cell culture supernatants (CS) and allantoic fluid (AF) were tested by a hemagglutination assay that used chicken erythrocytes, following the standard procedure [14]. Influenza type A was detected using a double antibody sandwich ELISA (NPA-ELISA) and an anti-NPA monoclonal antibody (ATCC n. HB65 H16-L10-4R5) as previously described [10]. The subtypes of the isolates were then determined from two multiplex RT-PCR assays [15]. Data of the strains analyzed in this study, including production phases, gross lesions, the presence of other pathogens and sequenced genes, are presented in Table 1.

**Table 1:** Data on the Italian strains investigated in this study.

	**Strains**	**Year**	**Group**	**Prov**	**Region**	**Production phases**	**HA GeneAcc Number**	**NA**	**Gross lesions**	**Other pathogens**
1	62	1998	B	MN	Lombardia-N	Fattening	HQ709201	HQ709202	n.a.	
2	3592	1999	A	MN	Lombardia-N	Fattening	HQ660233	HQ658492	n.a.	
3	18	2000	A	FC	Emilia Romagna-N	Weaning	HQ709203	HQ709204	Pneumonia with purple areas of consolidation	
4	22530	2002	C	PV	Lombardia-N	Weaning	HQ658491	HQ660234	Pneumonia with purple areas of consolidation	PRSV
5	4675	2003	B	MN	Lombardia-N	Sows	HM996942	HM996957	Pneumonia	*P. multocida*, PRRSV, PCV2, *M. hyopneumoniae*
6	259543	2003	A	PV	Lombardia-N	Fattening	JN022470	JN022471	Pneumonia with purple areas of consolidation, pleuritis, interstitial oedema	*M. hyopneumoniae*
7	50568	2005	B	CR	Lombardia-N	Fattening	HQ660235	HQ660236	Pneumonia with grey areas of consolidation in the apical and cardiac lobes	
8	53991	2005	B	BS	Lombardia-N	Fattening	KF305975	KF305948		
9	203047	2005	B	BS	Lombardia-N	Fattening	KF305976	KF305949	Pneumonia with grey areas of consolidation in the apical and cardiac lobes, pleuritis	PRSV, PCV2
10	232134	2005	B	CR	Lombardia-N	Fattening	HQ660249	HQ660250	Pneumonia with grey areas of consolidation in the apical and cardiac lobes	
11	233139	2005	B	PU	Marche- C°	Weaning	HQ660251	HQ660252	Pneumonia with purple areas of consolidation	
12	267010	2005	B	VE	Veneto-N	Weaning	KF305977	KF305950	Interstitial pneumonia	PCV2
13	626/2	2006	B	CR	Lombardia-N	Fattening	HQ658489	HQ658490	Pneumonia with grey areas of consolidation in the apical and cardiac lobes	PCV2, *A. pleuropneumoniae 1*, *P. multocida*
14	20333	2006	B	CR	Lombardia-N	Piglet	KF305978	KF305951	Pneumonia with areas of consolidation in the cardiac lobes	
15	114347/1	2006	B	BG	Lombardia-N	Fattening	HQ658487	HQ660244	Pneumonia with grey areas of consolidation in the apical and cardiac lobes	
16	226846	2006	B	MN	Lombardia-N	Fattening	KF305979	KF305952	Pneumonia with grey areas of consolidation	PRSV, PCV2
17	269578	2006	B	MN	Lombardia-N	Fattening	KF305980	KF305953	Bronchitis and pneumonia with areas of consolidation	PRSV
18	29141	2008	B	BS	Lombardia-N	Fattening	KF305981	KF305954	Suppurative broncopneumonia with grey areas of consolidation in the apical lobes	*A. pyogenes*
19	196875	2008	C	CN	Piemonte-N	Weaning	KF305982	KF305955	Interstitial pneumonia with areas of consolidation in the apical lobe	
20	198260	2008	B	BS	Lombardia-N	Piglet	HQ660247	HQ660248	Interstitial pneumonia, white necrotic foci in the myocardium	ECMV
21	59209/2	2009	B	BS	Lombardia-N	Fattening	HQ660237	HQ658488	Pneumonia with grey areas of consolidation and pleuritis	*P. multocida*
22	70757	2009	B	BS	Lombardia-N	Fattening	HQ660238	HQ660239	Pneumonia with grey areas of consolidation and pleuritis	PCV2 , *P. multocida*
23	81062	2009	B	BS	Lombardia-N	Piglet	HQ660240	HQ660241	Pneumonia and fibrinous pleuritis and pericarditis	PRSV, *H. parasuis*
24	81226	2009	B	PR	Emilia Romagna-N	Weaning	HQ660242	HQ660243	Pneumonia with monolateral purple areas of consolidation	PRSV, *H. parasuis*
25	191985	2009	B	LO	Lombardia-N	Fattening	HQ660245	HQ660246	Pneumonia and fibrinous pleuritis	*A. pleuropneumoniae 1*
26	274298	2009	B	BG	Lombardia-N	Fattening	HQ709193	HQ709194	Pneumonia with purple and grey areas of consolidation, interstitial oedema	PCV2, *M. hyopneumoniae*
27	289700	2009	B	BS	Lombardia-N	Weaning	HQ709195	HQ709196	Pneumonia and fibrinous pleuritis	PRSV, *P. multocida*
28	310411	2009	C	BS	Lombardia-N	Fattening	KF305983	KF305956	Interstitial pneumonia	PRSV
29	320546	2009	B	MN	Lombardia-N	Fattening	HQ709197	HQ709198	Interstitial and fibrinous pneumonia	PRSV, PCV2, *P. multocida*
30	321986	2009	B	BS	Lombardia-N	Fattening	HQ709199	HQ709200	Pneumonia with purple areas of consolidation, fibrinous pleuritis and pericarditis	PRSV, PCV2, *H. parasuis*
31	38272	2010	B	BS	Lombardia-N	Weaning	JN596916	JN596917	Pneumonia with grey areas of consolidation in the apical lobes	PRSV, PCV2
32	58769	2010	C	VR	Veneto-N	Piglet	HM771276	HM771275	Pneumonia with areas of consolidation in the apical lobes, fibrinous pleuritis	*A. pleuropneumoniae 1*
33	63580	2010	C	BS	Lombardia-N	Weaning	KF305984	KF305957	Poly-serositis and catarrhal enteritis	PRSV, *H. parasuis*, *M. hyopneumoniae*
34	76687	2010	B	CR	Lombardia-N	Weaning	KF305985	KF305958	Pneumonia with purple areas of consolidation in the apical and cardiac lobes	PRSV, PCV2
35	85218	2010	B	BS	Lombardia-N	Weaning	KF305986	KF305959	Interstitial pneumonia	
36	116114	2010	D	MN	Lombardia-N	Fattening	CY067662	CY067664	Pneumonia with red areas of consolidation in the apical lobes, , interlobular oedema, and fibrinous pleuritis	*S. suis*
37	118616	2010	B	PC	Emilia Romagna-N	Weaning	JN596920	JN596921	Pneumonia with areas of consolidation in the apical lobes	PRSV, PCV2
38	149992	2010	B	BS	Lombardia-N	Fattening	JN596922	JN596923	Pneumonia and fibrinous pleuritis	PRSV, P. multocida
39	166015	2010	B	BS	Lombardia-N	Weaning	KF305987	KF305960	Suppurative broncopneumonia	*A. pyogenes*
40	170177	2010	B	CR	Lombardia-N	Weaning	KF305988	KF305961	Pneumonia and fibrinous pleuritis and pericarditis	PRSV, *H. parasuis*
41	195639	2010	C	CN	Piemonte-N	Weaning	KF305989	KF305962	Pneumonia with red areas of consolidation in the apical lobes, interstitial oedema	PRSV, PCV2
42	282964	2010	B	MN	Lombardia-N	Fattening	JN596924	JN596925	Pneumonia, fibrinous pleuritis, interstitial oedema	PRSV, *P. multocida*, *M. hyopneumoniae*
43	254261	2010	B	BS	Lombardia-N	Piglet	KF305990	KF305963	Nasal swabs , monitoring program	
44	16959	2011	B	MN	Lombardia-N	Weaning	KF305991	KF305964	Nasal swabs , monitoring program	
45	134110	2011	B	MN	Lombardia-N	Weaning	KF305992	KF305965	Interstitial pneumonia, catarrhal enetritis	*E. coli O159*
46	186822	2011	B	RA	Emilia Romagna-N	Fattening	KF305993	KF305966	Pneumonia with red areas of consolidation, pleuritis	PCV2
47	195399	2011	C	CR	Lombardia-N	Weaning	KF305974	KF305967	Nasal swabs , monitoring program	
48	274551	2011	C	CN	Piemonte-N	Fattening	KF305994	KF305968	Nasal swabs , monitoring program	
49	308725	2011	B	CN	Piemonte-N	Weaning	KF305995	KF305969	Pneumonia with grey areas of consolidation in the apical lobes	
50	315977	2011	B	MO	Emilia Romagna-N	Weaning	KF305996	KF305970	Pneumonia with purple areas of consolidation	PRSV, PCV2
51	329017	2011	C	BS	Lombardia-N	Weaning	KF305997	KF305971	Nasal swabs, monitoring program	
52	26654	2012	C	CR	Lombardia-N	weaning	KF305998	KF305972	Pneumonia with red areas of consolidation, fibrinous arthritis	PRSV, *H. parasuis*
53	107798	2012	C	BS	Lombardia-N	Weaning	KF305999	KF305973	Interstitial pneumonia	PRSV, *H. parasuis*

^+^Northern Italy, °Central Italy.

n.a. no data available.

### Genome sequencing

Viral RNA was extracted from AF and CS using Trizol reagent (Invitrogen, Carlsbad, CA, USA) according to the manufacturer’s protocol, then purified using the QIAamp® ViralRNA Mini Kit (Qiagen, Hilden, Germany) and amplified by the OneStep RT-PCR Kit (Qiagen, Hilden, Germany) [16].

The full length HA and NA genes were amplified using universal primers as described by Hoffman et al. [17]. Amplified products were then separated on agarose gels and purified with the Qiaquick® gel extraction kit (Qiagen, Inc, Valencia, CA, USA). Products were sequenced using the BigDye® Terminator Cycle Sequencing kit v1.1 (Applied Biosystems, Foster City, CA, USA). Both strands of the amplicons were sequenced with the same forward and reverse primers that were used for the amplification. The full-length HA and NA amplicons were also sequenced with internal primers that are described elsewhere [15] and resolved on an ABI 3130 DNA automatic sequencer (Applied Biosystems, Foster City, CA, USA). DNA sequences were combined and edited using the Lasergene sequencing analysis software package (DNASTAR, Madison, WI, USA). Multiple sequence alignments were made using ClustalW. Distance-based phylogenetic trees were generated using the MEGA5 software [18].

### Phylogenetic analysis and dataset preparation

The phylogenetic trees were constructed by the Neighbor-joining (NJ) method using the kimura-two-parameter model. The results were verified using maximum likelihood and maximum parsimony analysis, which showed similar topologies. Gene sequences of the Italian strains were compared with swine, avian and human influenza virus sequences stored at the Influenza Virus Resource at the National Center for Biotechnology information (NCBI) [19].

For the evolutionary analyses, two data sets were prepared using the full-length sequences available; 1) the H1 gene with 120 HA sequences and 2) the N2 gene, with 161 NA sequences. Each data set was first aligned by ClustalW and then further adjusted manually in BioEdit [20].

### Phylogenetic inference, estimation of nucleotide substitution rate and times to common ancestors

Distance-based phylogenetic trees were constructed with the Maximum Likelihood (ML) method according to the general time-reversible (GTR) model of base substitution, using PhyML v.3.0 [21] and MEGA5 software [18]. The results were verified by NJ and maximum parsimony analysis, which showed similar topologies.

For the molecular clock analysis, the best-fit model of nucleotide substitution was determined for each data set using the jModelTest v.0.1.1 [22]. All the models were compared using two criteria, Akaike’s Information Criterion (AIC) and Bayesian information criterion (BIC). For HA and NA datasets, the GTR + G + I model resulted in the first model for AIC and the second for BIC and was then selected for both datasets. Four substitution rate categories were used with the gamma distribution parameter that was estimated to account for variable substitution rates among sites.

Rates of molecular evolution (i.e. nucleotide substitutions per site per year) and the time of the most recent common ancestor tMRCA were estimated for the HA and NA genes using the Bayesian Markov chain Monte Carlo (MCMC) approach as implemented in BEAST package v. 1.6.1 [23]. The marginal likelihoods of two different clock models, strict clock and relaxed uncorrelated lognormal clock (UCLD), were compared using a Bayes factor test for best fit [24,25]. Both datasets were analyzed using the GTR + G + Γ4 model of nucleotide substitution.

This test shows that for both genes, the UCLD model best fit the sequence data. The UCLD model was further tested with different demographic models (constant population size, exponential growth, and logistic growth and Bayesian skyline coalescent model). Convergence was assessed by effective sample size (ESS) values higher than 200 and 10% was discarded as burn-in. Uncertainty in parameter estimates was evaluated in the 95% highest posterior density (HPD 95%) interval. The Bayes Factor was used to select the model that better fit the data. Finally, Maximum Clade Credibility (MCC) trees were estimated from the posterior distribution of trees generated by BEAST using the Tree Annotator software v.1.6.1 after the removal of an appropriate burn-in. MCC tree was visualized using the Fig-Tree software v 1.3.1 [26], which allowed us to estimate the TMRCA of each individual node on the trees.

### Compilation of data sets and analysis of selection pressures

To better analyze selection pressure, HA and NA datasets were further divided into five sub-datasets according to the clades evidenced in the phylogenetic trees. Italian H1N2 strains were divided into four groups, named A, B, C and D, according to the HA phylogeny. Group A included strains with an H1 human-like; group B was formed by strains with an H1 human-like and a deletion of two amino acids in the HA protein; group C included strains with an H1 avian-like and group D only one strain closely related to 2009 H1N1 pandemic viruses (H1N1pdm).

Datasets used for analysis of selection pressures were the following: 1 - H1 avian-like (European (EU) H1N1 SIVs and group C); 2 - H1 human-like (EU H1N2 SIVs and group A and B); 3 - N2 (EU H1N2 SIVs and group A); 4 - N2 (human H3N2 influenza viruses); 5 - N2 (B and C strains with the NA related to human H3N2). To determine the selection pressure on the HA and NA genes, we estimated the rates of nonsynonymous (dN) and synonymous (dS) substitutions per site (ratio dN/dS) for each data set. We used the single likelihood ancestor counting (SLAC) and the fixed effects likelihood (FEL) methods that are available at the Datamonkey, which has an online version of the Hy-Phy package [27-29]. A best fit model for nucleotide substitution was estimated for each data set according to AIC. The dN/dS ratio was calculated using a codon model obtained by crossing MG94 and the best nucleotide model [30].

### Three-dimensional macromolecular structure (3D-MMS)

The three dimensional macromolecular structure (3D-MMS) of the HA proteins was predicted using the sequence-homology method that is based on sequences and structures released by the protein data bank (PDB) and visualized by Cn3D v4.3 software [31].

### Nucleotide sequence accession numbers

The GenBank numbers assigned to the gene sequences determined in this study are listed in Table 1.

All experimental researches performed in IZSLER are previously approved by a IZSLER Ethic committee and are performed according EU and national legislation, however in this study no experimental research is performed.

## Results

### Virus isolation and subtype determination

Virus was isolated on allantoic fluid and/or in MDCK and CACO-2 cell culture supernatants and then identified using an HA assay to test for hemagglutination and an NPA-ELISA to test for the presence of influenza A antigen. Isolated strains were then subtyped by multiplex RT-PCR that was specific for the HA and NA genes. We determined the complete sequences of the HA and NA genes from 53 Italian H1N2 strains that were isolated between 1998 and 2012 and included them in our evolutionary analysis. Year of isolation, geographical origin, gross lesions and production phase of these strains are reported in Table 1.

### Phylogenetic analysis of Italian H1N2 viruses

The NJ tree of the HA gene shows three highly significant clusters that corresponded to previously described clades: 1) EU H1N2 SIVs with a human-like H1, 2) EU H1N1 SIVs with an avian-like H1, and 3) the 2009 H1N1 pandemic viruses (H1N1pdm). Italian H1N2 strains could be subdivided into four groups according to the HA phylogeny: Group A included three strains isolated during 1998–2003 that had a human-like H1 that placed them in the EU H1N2 SIVs clade; Group B contained 38 strains, of which one was isolated in 1998 and 37 which were more recently isolated during 2003 to 2011. This group was placed in the same clade as group A (i.e., EU H1N2) but formed a separate sub-group that branched off from the 1998 isolate. Group B was characterized by the deletion of two amino acids in the HA protein at positions 146 and 147; Group C included 11 strains that have an avian-like H1 that placed them in the clade of EU H1N1 SIVs; Group D contained one reassorted H1N2 strain (A/Sw/It/116114/10) that was derived from the H1N1pdm [32], and which was grouped together with the H1N1pdm strains isolated from pig farms in Italy [33]. Italian strains divided by groups are shown in Table 1.

Phylogenetic analysis of the NA gene shows that the Italian strains could be divided into three different clades, EU H1N2 SIVs, EU H3N2 SIVs and human H3N2. The group A isolates together with a single group B strain that was isolated in 1998 were included in the EU H1N2SIVs clade. All the remaining strains in group B were included in the human H3N2 clade and the group C isolates had members in all three clades. Five strains belonged to human H3N2, five were part of EU H3N2SIVs and one was in EU H1N2SIVs. Group D was part of the EU H3N2SIV clade. Two additional files show the NJ trees of the HA and NA genes (see Additional files 1 and 2).

### Nucleotide substitution rates and times to common ancestors

**Figure 1:**
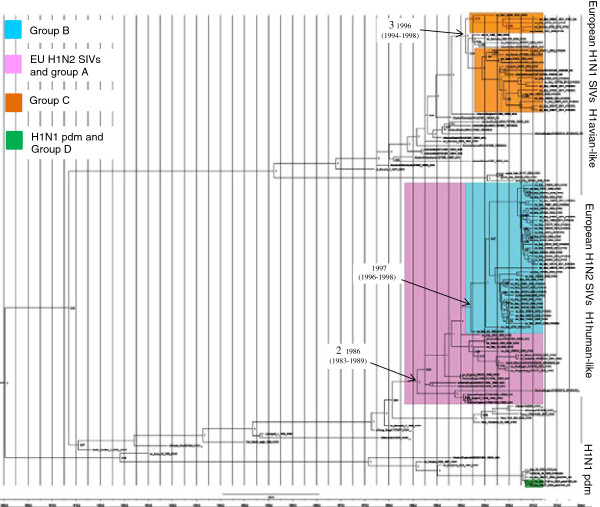
**Maximum clade credibility phylogeny and timing for the HA gene.** Phylogeny was estimated within an MCMC framework using the GTR + G + Γ4 nucleotide substitution model, the Bayesian skyline coalescent demographic model and the uncorrelated lognormal relaxed molecular clock. H1N2 strains are colored according to the phylogenetic classification of the HA gene. The most interesting internal nodes, which reconstructed common ancestors with 95% credibility intervals, are reported. Numbers 2 and 3 refer to the reassortment events described in Figure 3. For each node posterior probability is reported.

Based on the results of the Bayes factor, the model that best fit the data sets was the uncorrelated lognormal relaxed molecular clock with Bayesian skyline coalescent demographic model and the GTR + G + Γ_4_ model of nucleotide substitution. The MCC tree of the HA gene shows the same previously reported clades and groups (Figure 1).

**Figure 2:**
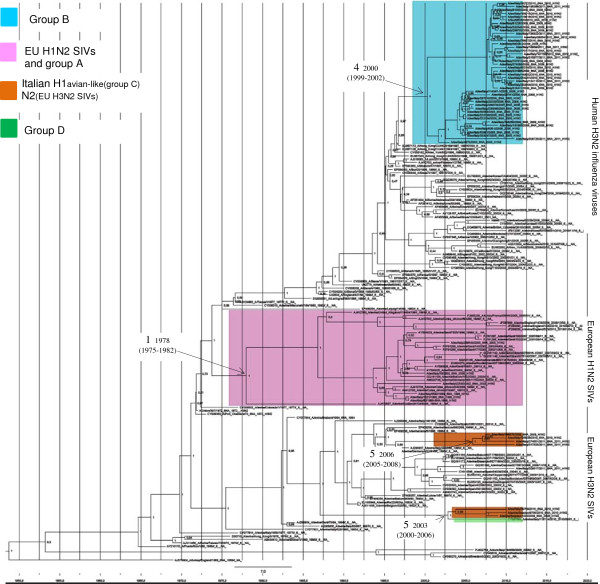
**Maximum clade credibility phylogeny and timing for the NA gene.** Phylogeny was estimated using the models reported in Figure 1. H1N2 strains are colored according to the phylogenetic classification of the HA gene. The most interesting internal nodes, which reconstructed common ancestors with 95% credibility intervals, are reported. Numbers 1, 4 and 5 refer to the reassortment events described in Figure 3. For each node posterior probability is reported.

The MCC tree of the NA gene shows the same three clades as the NJ tree: EU H1N2 SIVs, EU H3N2 SIVs and human H3N2 (Figure 2). The Italian strains were distributed along the three clades as described above. Interestingly, the NA gene of group B, except A/sw/It/62/98, clustered with human H3N2 IVs. BLAST analysis shows that the greatest similarity (from 97.1% to 95.1%) was to A/Hong Kong/CUK20199/97 H3N2. In general, the human H3N2 NA gene forms a seasonal phylogenetic cluster [34] and group B clustered with human H3N2 viruses from the 1997–1998 seasons. The eleven strains of group C were divided into three groups based on the sequence of the NA gene. The first group included five strains that show an uncommon pattern that arose from reassortment with an avian-like H1N1 HA SIVs and an EU H3N2 SIV-like NA. The second group contained five strains characterized by an avian-like H1 combined with an NA gene that was closely related to group B and human H3N2. The NA of the remaining group C strain (A/sw/It/22530/02) was related to EU H1N2 SIVs.

**Figure 3:**
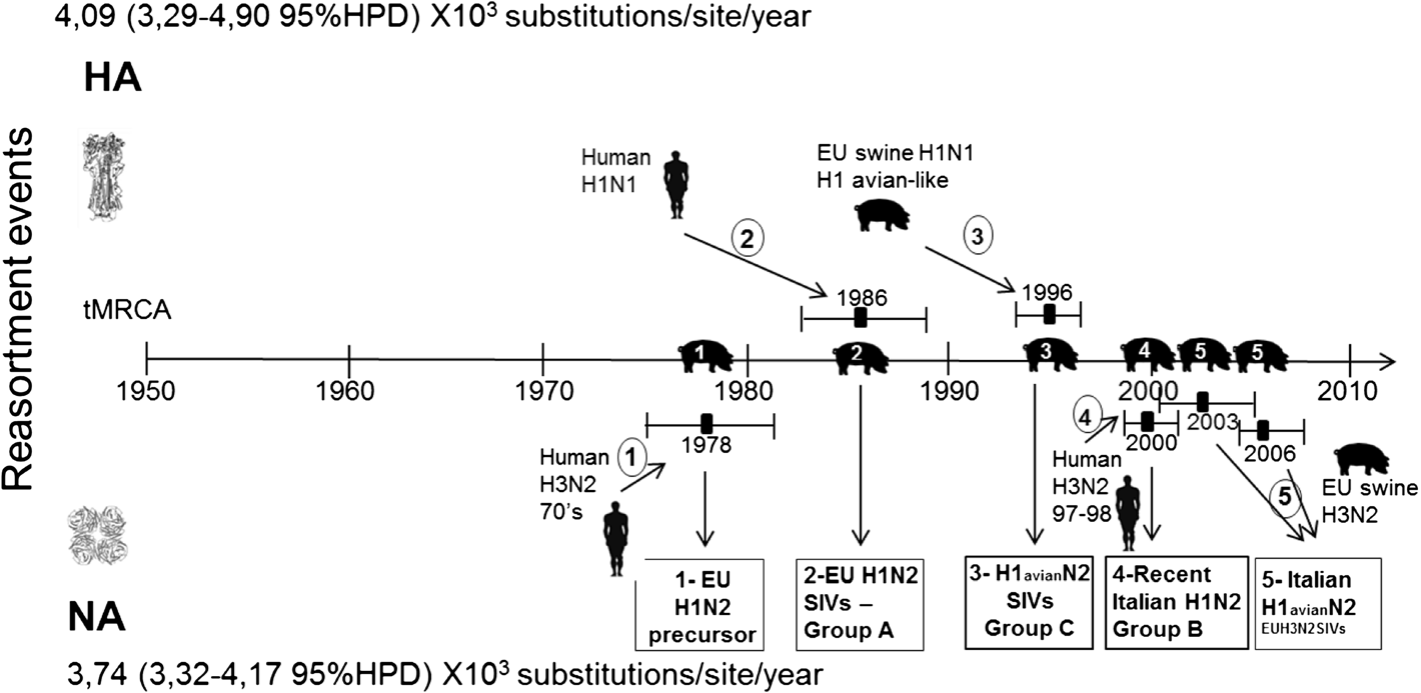
**Diagram of the reassortment events that generated the H1N2 SIVs types currently circulating in Europe.** The line represents time in years. The reassortment events that involve the HA gene are reported in the upper part of the diagram whereas those of the NA gene are in the lower part. The numbered reassortment events are the following: 1- the EU H1N2 SIVs was launched with the introduction of the NA gene from the Human H3N2 viruses; 2- the introduction of the HA gene from the human H1N1 viruses that generate the EU H1N2 SIVs and the Italian group A; 3- Introduction of an H1 avian like from the EU H1N1 SIVs in the H1N2 lineage that have given rise to the reassortant H1avian-likeN2 strains and the Italian group C; 4- Introduction of the NA gene from the more recent human H3N2 viruses that generate the Italian group B; 5- Introduction of the NA gene from the EU H3N2 SIVs that generate the reassortant H1N2(EU H3N2 SIVs) strains and Italian H1avian-likeN2(EU H3N2 SIVs). Mean estimates of TMRCAs with 95% credible intervals for each event are reported.

The highest number of substitutions was observed in the HA segment (4.09 × 10^3^ substitutions/site/year; 3, 29–4, 90 × 10^3^ 95% HPD), whereas in NA there were 3, 74 × 10^3^ (3, 32–4, 17 × 10^3^ 95% HPD). Based on these substitution rates, our tMRCA estimations suggest that a European H1N2 precursor acquired the NA gene from an old human H3N2 IVs by reassortment in 1978 (1975–1982; 95% HPD) and later obtained the HA gene by reassortment with the human H1N1 IVs in 1986 (1983–1989; 95% HPD). These two reassortment events would have given rise to the EU H1N2 SIVs and the Italian strains of group A. In addition, our analysis suggests three more reassortment events (named 3, 4 and 5) took place at different times between circulating swine and human influenza viruses: 3 – Italian viruses of group C, which introduced an avian-like H1 gene typical of the EU H1N1 SIVs in 1996 (1994–1998; 95% HPD) (Figure 1); 4 – Group B viruses were derived from a precursor that had a human-like H1, which is closely related to EU H1N1 SIVs, but was characterized by the deletion of two aa and which originated around 1997 (1996–1998; 95% HPD) (Figure 1). This precursor later acquired the NA gene from the Human H3N2 IVs in 2000 (1999–2002 95%HPD) (Figure 2). 5 – Some strains in group C acquired an NA gene derived from the EU H3N2 SIVs after the introduction of an avian-like H1. These viruses were divided into two subgroups, probably reflecting two different introductions, the first which occurred in 2003 (2000–2006 95% HPD) and the second which occurred in 2006 (2005–2008 95%HPD) (Figure 2). A summary of the different phylogenetic patterns of the HA and NA genes that highlights the reassortment events is shown in Figure 3.

### Molecular analysis and selection pressure

Comparisons of the deduced aa sequences of the HA gene of Italian strains and the European H1N2 SIVs stored in Influenza Virus Resource NCBI revealed the most interesting finding for group B. This group had a deletion of two aa at positions 146 and 147 in the HA protein, which were equivalent to residues 133 and 133A in H3 numbering [35]. The predicted 3D-MMS of the HA protein shows that these two residues are located in the membrane-distal globular portion of the molecule within the receptor-binding subdomain [35]. Three structural elements, the 190 helix (from R190 to R195), the 130 loop (from R133 to R138) and the 220 loop (from R217 to R230), form the sides of each binding site, which is made up of the conserved residues Y108, W167, H197 and Y209 (Y98, W153, H183 and Y195 in H3 numbering) [36]. The two aa deletions found in the Italian strains were located in the 130 loop. An additional file shows the predicted 3D-MMS of the HA and the three structural elements of the receptor binding subdomain (see Additional file 3).

Comparison of the deduced aa NA sequences Italian H1N2 strains revealed the presence of aa changes in the previously identified phylogenetically informative positions (PIP) and phylogenetically informative regions (PIR) [10].

The analysis of selection pressures revealed that most codons were subject to positive selection. For each dataset, values of mean dN/dS ratio (ω) and individual codons that were subject to positive selection are reported in Table 2. The ω values were below 1.0 for all datasets, indicating that there was no detectable positive selection on the gene as a whole. The ω of the human N2 dataset was the highest whereas the ω of the N2 of the recent Italian H1N2 SIVs dataset was the lowest. Site-by-site tests for positive selection helped to identify specific sites that were not detected by the global positive selection analysis. Results obtained using the SLAC and FEL methods were evaluated and SLAC analysis showed fewer sites under positive selection than the FEL analysis, for all datasets. Specifically, when FEL was used, 11 residues were identified that were positively selected for in the avian-like H1 dataset and six residues were found that were positively selected for in the human-like H1 dataset. Eight of eleven positively selected sites in avian like H1 and four of six in human-like H1 were located in the receptor binding site and some of them also formed part of the Sa, Sb and Ca antigenic sites [37]. No sites that are involved in receptor binding specificity [38] were under positive selection in either H1 dataset.

**Table 2:** Selection pressure and positively selected sites in the HA and NA genes from viruses from different datasets.

**Gene**	**Number of sequences**	**Length of alignment (aa)**	**Mean dN/dS**	**Positively selected sites**		**Domain**^**1**^	**Antigenic site**^**2**^
			**(95% CI)**	***P*** **= 0.1**			
				**SLAC**	**FEL**		
H1 avian-like	61	539	0.208 (019–0.22)	116	116	HA1 - E	
				137	137	HA1 -RBD	
					152	HA1 -RBD	
				159	159	HA1 -RBD	Ca
				172	172	HA1 -RBD	Sa
					185	HA1 -RBD	Ca
					213	HA1 -RBD	Sb
					232	HA1 -RBD	
				239	239	HA1 -RBD	
					392	HA2 - F	
					399	HA2 - F	
H1 human-like	65	555	0.225 (0.21-0.24)	102	102	HA1 - E	
					146	HA1 -RBD	
					158	HA1 -RBD	Ca
					213	HA1 -RBD	Sb
				271	271	HA1 -RBD	
				550	550	HA2 - F	
N2 EU sw H1N2	84	469	0.185 (0.17-0.20)		358	PIR H'	
					381		
					455		
N2 recent Italian H1N2	42	461	0.181 (0.15-0.22)	0	0		
N2 human H3N2	240	459	0.265 (0.24-0.29)		43	PIR A'	
				151	151	NA head domain	
				221	221	Antibody binding	
				267			
					339	PIR F'	Antigenic site
				370	370		Antigenic site

1 → HA domains: E- vestigial esterase, RBD – Receptor binding domain, F – membrane fusion subdomain in the HA2 subunit [38]. NA domains: PIR – phylogenetically important regions described by Fanning et al. [39], NA head domain and antibody binding positions were previously reported by Gulati et al. [40].

2 → Antigenic sites in HA: Ca, Sa, Sb described by Caton et al. [37] and for H1N1pdm by Xu et al. [41]. Antigenic sites in NA: described by Colman et al. [42] and Air et al. [43].

Numbering is based on defining the first amino acid of the open reading frame as amino acid n. 1.

Several sites under positive selection were found in both the N2 EU H1N2 SIVs and human N2 H3N2 datasets. Six sites in the human N2 of the H3N2 virus were positively selected for, and three sites in the N2 of EU H1N2 SIVs were positively selected for. Four of the six sites identified in the human N2 were located in important domains (phylogenetically important regions, NA head and antibody binding domains). In contrast, we found no sites in the N2 of recent Italian strains that were under positive selection. For each dataset, the selection pressure, the positively selected sites and their location in the protein are reported in Table 2.

## Discussion

This study focused on the evolutionary dynamics of the H1N2 subtype isolated from Italian swine, beginning in 1998 and continuing into 2012. To perform a phylogenetic and molecular analysis of the HA and NA genes of 53 Italian H1N2 strains, we compared their gene sequences with the Influenza Virus Resource NCBI sequence collection of swine influenza viruses and influenza viruses of human and avian origin. Our analyses revealed a clear difference between Italian strains of group A, which were closely related to the EU H1N2 SIVs, and group B, which had a different HA-NA combination. This difference was characterized by an HA that derived from an H1N2 strain that was isolated in Italy in 1998 that had two aa deletions within the receptor binding site of the HA protein (Residues 146 and 147, which are equivalent to 133 and 133A in H3 numbering), and an NA gene that was closely related to the human H3N2 viruses of 1997. To our knowledge, a single aa deletion at position 147 has been observed in 4/285 (1.6%) of H1 European SIVs sequences available in GenBank, whereas the deletion of two aa was only found in Italian strains. In addition, we identified three different groups of strains that arose from reassortment in group C, which are characterized by an avian-like H1 that is combined with the N2 of one of the following clades: EUH1N2SIVs, EU H3N2SIVs or Human H3N2SIVs. Interestingly, strains with an N2 protein that is closely related to EU H1N2SIVs apparently have not circulated in Italy since 2003, because all the strains isolated after 2003 contained NA proteins that were closely related to human H3N2 or EU H3N2SIVs.

The time-scaled phylogeny of the HA protein revealed that Italian strains could be segregated into four different groups. The majority of them was in groups A and B, and had a human-like H1 antigen, which probably originated in 1986. Group B however, formed a sub-group that was characterized by a two aa deletion in the HA protein. In contrast to A and B, the third group of 11 isolates (Group C) was characterized by an avian-like H1, which probably originated in 1996. Group D had only one strain that had an H1 characteristic of the 2009 pandemic strain. The analysis of the dated NA phylogeny suggests that the observed Italian swine strains arose from a series of reassortment events. In particular, whereas the oldest strains in group A have an NA protein that originated from human H3N2 viruses in the late 1970s, the isolates in the more recent group B, excluding one strain isolated in 1998, are characterized by the two aa deletion in the H1 protein, and a different human N2. This N2 probably resulted from reassortment with a more recent H3N2 virus, which was circulating among humans and took place in 2000 or later. The analysis of group C isolates was more complex because the NA protein of five of these strains was derived from EU swine H3N2, for one it was derived from EU swine H1N2, and for the other five, similar to the group B isolates, the N2 was derived from human H3N2. Furthermore, the five strains characterized by the avian-like H1 and swine H3N2 NA protein segregated into two groups, suggesting that at least two other reassortment events took place in 2003 and 2006. Finally for group D, the virus has the H1(2009)pdm and an NA protein derived from swine H3N2, which originated in 2003.

To better characterize the effect of the two aa deletion found in the Group B H1 gene, the 3D-MMD of the HA protein was predicted. The region of HA that contains the receptor binding residues is located at the membrane –distal tip of each monomer of the HA trimer. This binding site is flanked by three elements. The 220 and 130 loops contain amino acids that interact with sialic acid or internal sugars of the glycan chain. The 190 helix forms the membrane-distal region of the site and includes residues that have the potential to contact the sialic acid or internal glycans on the receptor [44]. The two deleted amino acids are at position 133 and 133A (H3 numbering), which is located in the 130 loop and therefore, could affect virus interactions with cell surface receptors. In order to better investigate the effects of these interesting deletions on the receptor interactions further studies are required.

Theoretically, reassortment events between human and swine influenza viruses might frequently occur but fail to persist in the pig population [45]. Indeed, new virus strains with different antigenic characteristics may be at an advantage or disadvantage compared to well-adapted, established viruses already circulating in pigs. Those at an advantage could persist in pigs and, following adaptation, could be associated with clinical disease [46]. The successful transmission of Influenza A virus (IAV) depends on a specific gene constellation [46] and a better balanced HA-NA gene combination [47]. HA and NA proteins both recognize sialic acid but with conflicting activities, and a balance of HA and NA protein activity is essential to ensure efficient viral replication [47]. While a virus is adapting to a new host, possibly by improving its transmission efficiency, a functional balance between HA binding and NA enzymatic activity may occur [48]. The emergence and persistence of group B strains suggest that a particular gene constellation and an optimum balance between HA and NA activity contributes to its efficient replication and successful transmission among pigs. Another interesting group that was formed by strains of group C with an avian-like H1 has been increasing in the last few years. Indeed out of eleven C isolates, eight were isolated in the last three years and 50% of the H1N2 strains isolated in 2011–2012 also contained this hemagglutinin variant. These data suggest that, together with the previously established group B, there is now a persistence of group C strains and both B and C have probably replaced the EU H1N2 SIVs in our country, because they have not been isolated since 2003.

Positive selection for the full length HA and NA sequences of the H3N2 and H1N1 viruses was previously reported [49-52]. However, there was a lack of detailed description of these analyses in swine influenza viruses because the two swine lineages were not differentiated. Although Li et al. [51] distinguished between them, the European SIVs were analysed as a unique dataset and differences between the HA protein of viruses circulating in Europe (EU H1 avian origin SIVs and EU H1 human origin SIVs) were not considered. In our study, five different datasets were taken into account: for HA - European avian-like H1 and human-like H1 and for NA – three datasets that corresponded to different clusters in the NA tree. These were the N2 of EU H1N1 SIVs, recent Italian SIVs and recent human H3N2 IVs. Swine influenza virus is considered to be under weak selection pressure by the host’s immune system [9] and this was confirmed by the lower ω values we observed for the different datasets (Table 2). Selection pressure analyses of Italian strains, except for Italian B and C strains with the NA gene related to human H3N2, were performed together with the EU SIVs because of the observed close genetic relationship. The NA gene of the remaining Italian strains is derived from the recent human H3N2 strains; it is found only in Italy and therefore was analyzed separately. Interestingly, for this dataset the global ω value was lower than that of human viruses and similar to the EU SIVs according to a host-specific evolution of influenza virus genes [53].

Because of the short average life span of pigs, swine influenza virus evolution may be determined to only a limited extent by immune pressure, which is the driving force of antigenic drift of influenza viruses in humans [9]. Human influenza viruses require frequent antigenic changes of HA to ensure that a sufficiently large pool of immunologically susceptible hosts is available. The situation for swine influenza viruses is different due to a continuous renewal of the susceptible pig population since the major part of pigs are killed at the age of 6 months (up to 8 months in Italy) consequently limiting the increase of immune pressure. Only adult sows used for pig breeding have a long life, experiencing more than one influenza season and could create some degree of immune pressure. Influenza vaccination of pigs is applied in Europe using inactivated, bivalent vaccines, that are used mainly in gilts and sows; however this vaccination is used in a low number of breeding farms (below 30%) in Italy (G Alborali, unpublished observations). Considering the limited vaccination in Italy and the continual supply of non-immune animals, we could hypothesize that vaccination does not play an important role in the evolution of Italian swine influenza viruses.

Site-by-site analysis revealed that for both HA types, several sites were under positive selection. Some sites were located in the receptor binding site and some of them formed part of the Sa, Sb and Ca antigenic sites. For NA, the N2 of human origin was subject to the strongest positive selection. Four of the six sites identified were located in important domains (phylogenetically important regions, NA head and antibody binding domains). These results demonstrate that although the selection pressure on SIVs is weak, selection has influenced the evolution of the virus, leading to amino acid substitutions at several antigenic sites.

Continuous monitoring of the genetic content of circulating IAV in order to detect new reassortment events, and studies that define the processes involved in viral reassortment are essential if we are to understand how pandemic IAV arise. Understanding IAV evolution and adaptation to various hosts will also provide information on their ability to cross host barriers and develop into pandemic strains.

## Abbreviations

EU H1N2SIVs: European H1N2 swine influenza viruses; HA: Hemagglutinin; NA: Neuraminidase; aa: Amino acid; SIVs: Swine influenza viruses; TMRCA: Time to the most recent common ancestor; GTR: General time-reversible; UCLD: Uncorrelated lognormal clock; IAV: Influenza a viruses; NJ: Neighbor-joining; pdm: Pandemic; SLAC: Single likelihood ancestor counting; dN: Non synonymous; dS: Synonymous; ML: Maximum likelihood; MCMC: Bayesian Markov chain Monte Carlo; MCC: Maximum Clade credibility; ESS: Effective sample sizes; FEL: Fixed effects likelihood; 3D-MMS: Three dimensional macromolecular structure; PDB: Protein data bank; AIC: Akaike’s information criterion; BIC: Bayesian information criterion.

## Competing interests

The authors declare that they have no competing interests.

## Authors’ contributions

AM conceived and designed the study, carried out molecular and evolutionary studies and selection pressure analysis, and drafted the manuscript; EG participated in the sequence alignment and evolutionary studies; ES and DL participated in the molecular studies and virus isolation; CC participated in virus isolation and characterization and collected anamnestic data; GZ and MC participated in the evolutionary studies and helped to draft the manuscript; PC participated in designing and coordinating the study, and helped to draft the manuscript. All authors have read and approved the manuscript.

## Supplementary Material

Additional file 1 **Phylogenetic tree of the HA gene.** Gene sequences of the Italian strains were compared with swine, avian and human influenza virus sequences stored at the Influenza Virus Resource at the National Center for Biotechnology information (NCBI). The unrooted tree was generated with the MEGA5 program using the Neighbor-Joining method. The evolutionary distances were computed using the Kimura 2-parameter method. Bootstrap values were calculated on 1000 replicates and only values higher than 70% are shown. Viruses used in this study are underlined. Click here for file

Additional file 2 **Phylogenetic tree of the NA gene.** Gene sequences of the Italian strains were compared with swine, avian and human influenza virus sequences stored at the Influenza Virus Resource at the National Center for Biotechnology information (NCBI). The unrooted tree was generated as described in Figure 1. Viruses used in this study are underlined. Click here for file

Additional file 3 **Predicted 3D-MMS of monomer of the H1 protein (a: lateral view and b: top view).** Amino acid residues R133 and 133A, which are deleted in the recent Italian H1N2 strains, are shown in red. Numbering is expressed in H3 numbering. The receptor-binding subdomain, which is located in the globular part of the molecule, and the three secondary structure units making up the site are shown in yellow [35,36]. Click here for file
