# Supplementary material for: Different evolutionary trends of swine H1N2 influenza viruses in Italy compared to European viruses
Source: Vet Res. 2013 Dec 1;44(1):112. doi: 10.1186/1297-9716-44-112 (PMC4176092; doi:10.1186/1297-9716-44-112)
Supplement: Additional file 1 — Phylogenetic tree of the HA gene. Gene sequences of the Italian strains were compared with swine, avian and human influenza virus sequences stored at the Influenza Virus Resource at the National Center for Biotechnology information (NCBI). The unrooted tree was generated with the MEGA5 program using the Neighbor-Joining method. The evolutionary distances were computed using the Kimura 2-parameter method. Bootstrap values were calculated on 1000 replicates and only values higher than 70% are shown. Viruses used in this study are underlined. [file 1297-9716-44-112-S1.docx]

sw Italy 170177 2010 H1N2HA

sw Italy 16959-2 2011 H1N2HA

sw Italy 315977 2011 H1N2HA

sw Italy 289700 2009 H1N2

sw Italy 321986 2009 H1N2

sw Italy 38272 2010H1N2

sw Italy 81062 2009 H1N2

sw Italy 81226 2009 H1N2

sw Italy 166015 2010 H1N2HA

sw Italy 76687 2010 H1N2HA

sw Italy 186822 2011 H1N2HA

sw Italy 85218 2010 H1N2HA

sw Italy 320546 2009 H1N2

sw Italy 149992 2010 H1N2

sw Italy 254261 2010 H1N2HA

sw Italy 282964 2010 H1N2

sw Italt 134110 2011 H1N2HA

sw Italy 118616 2010 H1N2

sw Italy 70757 2009 H1N2

sw Italy 59209-2 2009 H1N2

sw Italy 274298 2009 H1N2

sw Italy 4675 2003 H1N2

sw Italy 308725 2011 H1N2HA

sw Italy 114347 2006 H1N2

sw Italy 29141 2008 H1N2HA

sw Italy 198260 2008 H1N2

sw Italy 191985 2009 H1N2

sw Italy 266846 2006 H1N2HA

sw Italy 269578 2006 H1N2HA

sw Italy 53991 2005 H1N2HA

sw Italy 203047 2005 H1N2HA

sw Italy 626 2006 H1N2

sw Italy 267010 2005 H1N2HA

sw Italy 50568 2005 H1N2

sw Italy 232134 2005 H1N2

sw Italy 233139 2005 H1N2

sw Italy 62 1998 H1N2

sw Italy 3592 1999 H1N2

sw Italy 259543 2003 H1N2

A/swine/Bakum/1832/2000 2000// 4HA

sw Bakum 1832 2000 H1N2

sw Italy 18 2000 H1N2

sw Groitzsch IDT6016-2 2007 H1N2

sw Kitzen IDT6142 2007 H1N2

A/swine/Granstedt/IDT3475/2004 2004/08/26 HA

sw Cloppenburg IDT4777 2005 H1N2

sw Doetlingen IDT4735 2005 H1N2

A/swine/England/690421/1995 1995// 4HA

sw England 690421 1995 H1N2

A/swine/England/1382/2010 2010/04/13 HA

A/swine/England/72685/1996 1996// 4HA

A/swine/England/17394/1996 1996//4 HA

sw England 72685 1996 H1N2

A/swine/England/438207/1994 1994// 4HA

A/swine/Scotland/410440/1994 1994// 4HA

A/Berlin/13/2006(H1N1))

A/Norway/167/2008(H1N1))

New Caledonia 20 1999 H1N1

New York 294 2003 H1N2

Texas UR06-0420 2007 H1N1

hu Memphis 7 1980 H1N1

A/Hong Kong/117/1977(H1N1))

A/Memphis/1/1978(H1N1))

Leningrad 1 1954 H1N1

Fort Worth seg6 1950 H1N1

A/Puerto Rico/8/1934(H1N1))

South Carolina 1 1918 H1N1

sw Iowa 15 1930 H1N1

A/swine/England/283902/1993 1993// 4HA

sw Kansas 3228 1987 H1N1

sw Maryland 2323 1991 H1N1

Sw Italy 116114 2010 pdmH1N2 HA

sw Italy 85437 2009 pdmH1N1 HA

California 04 2009 H1N1pdm

Italy 05 2009 H1N1pdm

sw Italy 290271 2009 pdmH1N1 HA

goose Italy 296426 2003 H1N1

goose Italy 61117 2004 H1N1

duck Italy 281904 2006 H1N1

dk Bavaria 1 1977 H1N1

A/swine/England/WVL15/1997 1997//4HA

A/swine/England/WVL7/1992 1992//4HA

A/swine/Spain/WVL6/1991 1991// 4HA

sw Germany 8533 1991 H1N1

A/swine/Italy/670/1987 1987// 4HA

A/swine/Italy/671/1987 1987// 4HA

A/swine/Belgium/1/1998 1998// 4HA

A/swine/Denmark/WVL9/1993 1993//4 HA

A/swine/Scotland/WVL17/1999 1999// 4HA

A/swine/France/WVL13/1995 1995//4HA

A/swine/Netherlands/3/1980 1980// 4HA

A/swine/Netherlands/12/1985 1985// 4HA

A/swine/Parma/1997 1997// 4HA

A/swine/England/1389/2010 2010/04/15 HA

sw Sweden 1021 2009 H1N2

sw Sweden 9706 2010 H1N2

sw Italy 58769 2010 H1N2

sw Italy 266991 2011 H1N1 HA

sw Germany SEk1178 2000 H1N2

sw IV 1455 1999 H1N1

A/swine/Spain/50047/2003 2003// 4HA

sw Haseluenne IDT2617 2003 H1N1

sw Italy 22530 2002 H1N2

A/swine/Spain/51915/2003 2003// 4HA

sw Spain 51915 2003 H1N1

sw Greven IDT2889 2004 H1N1

sw Italy 310411 2009 H1N2HA

sw Italy 206127 2011 H1N1HA

sw Italy 329017 2011 H1N2HA

sw Italy 26654 2012 H1N2HA

sw Italy 196875 2008 H1N2HA

sw Italy 195639 2010 H1N2HA

sw Italy 274551-4 2011 H1N2HA

sw Italy 274551-6 2011 H1N2HA

A/swine/Germany/SIV04/2008 2008/06/ HA

A/swine/Hungary/19774/2006 2006/09/ HA

sw Italy 107798 2012 H1N2HA

sw Italy 195399 2011 H1N2HA

sw Italy 63580 2010 H1N2HA

sw Spain 53207 2004 H1N1

100

100

100

100

93

100

100

98

100

100

91

99

100

90

75

87

93

79

100

100

100

100

100

100

100

100

96

99

100

98

100

100

97

100

100

75

100

91

100

100

100

80

89

97

100

97

87

89

92

91

100

94

100

99

99

96

99

81

86

89

100

0.05

European H1 human-like SIVs

European H1 avian-like SIVs

Human H1N1 pdm

Human H1N1 IVs

Avian H1N1 IVs

Recent Italian H1N2 strains
