# Supplementary material for: Different evolutionary trends of swine H1N2 influenza viruses in Italy compared to European viruses
Source: Vet Res. 2013 Dec 1;44(1):112. doi: 10.1186/1297-9716-44-112 (PMC4176092; doi:10.1186/1297-9716-44-112)
Supplement: Additional file 2 — Phylogenetic tree of the NA gene. Gene sequences of the Italian strains were compared with swine, avian and human influenza virus sequences stored at the Influenza Virus Resource at the National Center for Biotechnology information (NCBI). The unrooted tree was generated as described in Figure 1. Viruses used in this study are underlined. [file 1297-9716-44-112-S2.docx]

A/sw/Italy/282964/2010 6NA 2010 H1N2

A/sw/Italy/134110/2011 6NA 2011 H1N2

A/sw/Italy/254261/2010 6NA 2010 H1N2

A/sw/Italy/85218/2010 6NA 2010 H1N2

A/sw/Italy/321986/2009 6NA 2009 H1N2

A/sw/Italy/310411/2009 6NA 2009 H1N2

A/sw/Italy/149992/2010 6NA 2010 H1N2

A/sw/Italy/118616/2010 6NA 2010 H1N2

A/sw/Italy/289700/2009 6NA 2009 H1N2

A/sw/Italy/320546/2009 6NA 2009 H1N2

A/sw/Italy/81062/2009 6NA 2009 H1N2

A/sw/Italy/166015/2010 6NA 2010 H1N2

A/sw/Italy/81226/2009 6NA 2009 H1N2

A/sw/Italy/59209-2/2009 2009 H1N2

A/sw/Italy/70757/2009 6NA 2009 H1N2

A/sw/Italy/274298/2009 6NA 2009 H1N2

A/sw/Italy/107798/2012 H1N2 NA

A/sw/Italy/315977/2011 6NA 2011 H1N2

A/sw/Italy/170177/2010 6NA 2010 H1N2

A/sw/Italy/16959/2011 6NA 2011 H1N2

A/sw/Italy/38272/2010 6NA 2010 H1N2

A/sw/Italy/195399/2011 6NA 2011 H1N2

A/sw/Italy/76687/2010 6NA 2010 H1N2

A/sw/Italy/186822/2011 6NA 2011 H1N2

A/sw/Italy/26654/2012 6NA 2012 H1N2

A/sw/Italy/308725/2011 6NA 2011 H1N2

A/sw/Italy/4675/2003 6NA 2003 H1N2

A/sw/Italy/198260/2008 6NA 2008 H1N2

A/sw/Italy/191985/2009 6NA 2009 H1N2

A/sw/Italy/626-2/2006 2006 H1N2

A/sw/Italy/203047/2005 6NA 2005 H1N2

A/sw/Italy/53991/2005 6NA 2005 H1N2

A/sw/Italy/232134/2005 6NA 2005 H1N2

A/sw/Italy/233139/2005 6NA 2005 H1N2

A/sw/Italy/114347-1/2006 2006 H1N2

A/sw/Italy/29141/2008 6NA 2008 H1N2

A/sw/Italy/50568/2005 6NA 2005 H1N2

A/sw/Italy/267010/2005 6NA 2005 H1N2

A/sw/Italy/20333/2006 6NA 2006 H1N2

A/sw/Italy/226846/2006 6NA 2006 H1N2

A/sw/Italy/269578/2006 6NA 2006 H1N2

EU857172 A/Hong Kong/CUHK20199/1997 1997/07/03 6 (NA)

EU857126 A/Hong Kong/CUHK12563/1997 1997/06/02 6 (NA)

CY008182 A/New York/502/1998 1998/03/20 6 (NA)

EU857303 A/Hong Kong/CUHK50552/1998 1998/12/21 6 (NA)

EU852000 A/New York/06/2008 2008/02/13 6 (NA)

HQ315824 A/Stockholm/4/2010 2010/07/08 6 (NA)

EU103978 A/California/07/2004 2004// 6 (NA)

EF455564 A/swine/Guangdong/01/2005 2005// 6 (NA)

AJ489848 A/England/1/02 2002 2002// 6 (NA)

CY037345 A/Genoa/1/2002 2002/12/02 6 (NA)

DQ469984 A/swine/Manitoba/12707/2005 2005// 6 (NA)

CY045561 A/swine/Kansas/015252/2009 2009// 6 (NA)

HM461772 A/swine/Iowa/02039/2008 2008// 6 (NA)

DQ469976 A/swine/British Columbia/28103/2005 2005// 6 (NA)

JF812297 A/swine/Iowa/A01049031/2010 2010/11/16 6 (NA)

AJ412703 swine/Finistere/127/99 1999// 6 (NA)

EF584360 A/Moscow/41/1997 1997// 6 (NA)

EF584343 A/Bur/23/1996 1996// 6 (NA)

CY061743 A/swine/Hong Kong/2314/2009 2009/10/22 6 (NA)

GQ229370 A/swine/Hong Kong/NS623/2002 2002/07/09 6 (NA)

EF556202 A/swine/Guangxi/17/2005 2005// 6 (NA)

EF556204 A/swine/Hainan/1/2005 2005// 6 (NA)

GQ229346 A/swine/Hong Kong/294/2009 2009/02/23 6 (NA)

AF251404 A/Swine/Nebraska/209/98 1998// 6 (NA)

AF251412 A/Swine/Iowa/533/99 1999// 6 (NA)

EU798830 A/swine/Korea/CAN04/2005 2005// 6 (NA)

AF455695 A/Swine/Iowa/930/01 2001// 6 (NA)

AF455698 A/Swine/Illinois/100084/01 2001(NA)

AY129157 A/Swine/Korea/CY02/2002 2002// 6 (NA)

EU798824 A/swine/Korea/Asan04/2006 2006// 6 (NA)

EF584376 A/Eng/23/1996 1996// 6 (NA)

EF584370 A/Parma/2/1997 1997// 6 (NA)

CY085584 A/swine/Hong Kong/1102/2003 2003/08/14 6 (NA)

CY085488 A/swine/Hong Kong/NS13/2002 2002/01/21 6 (NA)

CY085632 A/swine/Hong Kong/NS157/2004 2004/02/23 6 (NA)

GQ229322 A/swine/Hong Kong/554/2003 2003/04/24 6 (NA)

CY036833 A/Siena/1/1991 1991/12/17 6 (NA)

U42775 A/Netherlands/938/92 1992// 6 (NA)

DQ508851 A/Leningrad/360/1986 1986// 6 (NA)

CY035208 A/Siena/4/1990 1990/01/08 6 (NA)

CY035200 A/Siena/3/1988 1988/02/05 6 (NA)

U42634 A/England/427/88 1988// 6 (NA)

EF409254 A/swine/Leipzig/145/92 1992// 6 (NA)

A/sw/Italy/18/2000 6NA 2000 H1N2

AY590829 A/swine/Gent/7625/1999 1999// 6 (NA)

GQ161106 A/swine/Bakum/1832/2000 2000// 6 (NA)

GQ161165 A/swine/Granstedt/IDT3475/2004 2004/08/26 6 (NA)

GQ161142 A/swine/Groitzsch/IDT6016-1/2007 2007/03/22 6 (NA)

GQ161146 A/swine/Kitzen/IDT6142/2007 2007/05/11 6 (NA)

A/sw/Italy/259543/2003 6NA 2003 H1N2

FJ791299 A/swine/Gent/143/2002 2002/08/09 6 (NA)

AY590826 A/sw/Gent/24/00 2000// 6 (NA)

AY590828 A/sw/Gent/108/01 2001// 6 (NA)

AM920738 A/swine/Germany/SEk1178/2000 2000// 6 (NA)

A/sw/Italy/22530/2002 6NA 2002 H1N2

AJ412704 A/swine/Cotes dArmor/2433/98 1998// 6 (NA)

AJ412705 A/swine/Cotes dArmor/790/97 1997// 6 (NA)

A/sw/Italy/3592/1999 6NA 1999 H1N2

A/sw/Italy/62/1998-6NA-1998 H1N2

A/sw/Italy/1521/1998 6NA 1998 H1N2

FJ791297 A/swine/Gent/100/2007 2007/09/11 6 (NA)

EU924273 A/swine/Nordkirchen/IDT1993/2003 2003/04/ 6 (NA)

AJ412700 A/swine/United Kingdom/119404/1991 1991// 6 (NA)

AJ412702 A/swine/Cotes dArmor/604/99 1999// 6 (NA)

FJ985238 A/turkey/France/05045/2005 2005/01/ 6 (NA)

JF298000 A/swine/England/523/2010 2010/01/04 6 (NA)

JF290393 A/swine/England/1382/2010 2010/04/13 6 (NA)

JF297999 A/swine/England/1428/2009 2009/12/08 6 (NA)

AJ412697 A/swine/Scotland/410440/94 1994// 6 (NA)

CY009310 A/swine/Wisconsin/194/1980 1980// 6 (NA)

AB124662 A/Texas/1/1977 1977// 6 (NA)

CY009350 A/Port Chalmers/73 6NA 1973 H3N2

A/Udorn/307/1972 6NA 1972 H3N2

CY009302 A/swine/Colorado/1/1977 1977// 6 (NA)

AB434410 A/swine/Ehime/1/1980 1980// 6 (NA)

D00715 A/swine/Hong Kong/3/1976 1976// 6 (NA)

AY210115 A/PuertoRico/1/68 1968// 6 (NA)

CY077914 A/swine/Italy/1850/1977 1977// 6 (NA)

CY077921 A/swine/Italy/526/1985 1985// 6 (NA)

AJ293934 A/swine/Gent/70/84 1984// 6 (NA)

CY077944 A/swine/Brabant/1984 6NA 1984

AJ293935 A/swine/Italy/636/87 1987// 6 (NA)

A/swine/Sweden/9706/2010 H1N2 NA

A/swine/Sweden/1021/2009 H1N2 NA

A/sw/Italy/58769/2010 6NA 2010 H1N2

CY067664 A/swine/Italy/116114/2010 2010/06/01 6 (NA)

AJ293936 A/swine/Italy/1461/96 1996// 6 (NA)

A/sw/Italy/196875/2008 6NA 2008 H1N2

A/sw/Italy/195639/2010 6NA 2010 H1N2

A/sw/Italy/274551/2011 6NA 2011 H1N2

A/sw/Italy/317525/2011 6NA 2011 H1N2

AJ293937 A/swine/Italy/1510/98 1998// 6 (NA)

EF409256 A/swine/Jena/5/1996 1996// 6 (NA)

EF409258 A/swine/Bakum/8602/99 1999// 6 (NA)

AM920724 A/swine/Germany/Vi3161/95 1995// 6 (NA)

CY009374 A/swine/Spain/33601/2001 2001// 6 (NA)

AJ311455 A/swine/Belgium/220/92 1992// 6 (NA)

EU163949 A/swine/Re220/92hp 1992// 6 (NA)

EF409255 A/swine/Bakum/909/93 1993// 6 (NA)

EF409257 A/swine/Lohne/1/97 1997// 6 (NA)

AM920743 A/swine/Germany/S043/2002 2002// 6 (NA)

CY009382 A/swine/Spain/39139/2002 2002// 6 (NA)

A/sw/Italy/329017/2011 6NA 2011 H1N2

GQ161148 A/swine/Damme/IDT5673/2006 2006/11/16 6 (NA)

EU163948 A/swine/Bakum/IDT1769/2003 2003// 6 (NA)

GQ161100 A/swine/Bakum/IDT1769/2003 2003/03/07 6 (NA)

GQ161172 A/swine/Bissendorf/IDT1864/2003 2003/03/19 6 (NA)

CY010566 A/swine/Spain/54008/2004 2004// 6 (NA)

CY020503 A/swine/Spain/42386/2002 2002// 6 (NA)

EU053143 A/swine/Cloppenburg/IDT4777/2005 2005/12/13 6 (NA)

EU053135 A/swine/Doetlingen/IDT4735/2005 2005/12/07 6 (NA)

GQ161152 A/swine/Bondelum/IDT5959/2007 2007/02/28 6 (NA)

AJ574904 A/turkey/England/1969 6NA 1969// NA)

FJ432764 A/duck/Italy/194659/2006 2006// 6 (NA)

CY043818 A/mallard/Netherlands/1/2007 2007// 6 (NA)

CY060270 A/mallard/Sweden/3/2002 2002/10/20 6 (NA)

100

84

100

100

93

100

100

100

100

97

100

99

69

82

89

100

78

100

80

76

99

79

76

90

83

80

100

78

87

96

93

97

95

99

98

100

100

96

97

99

96

100

93

100

100

82

100

87

100

100

100

99

100

99

84

97

94

95

99

95

100

97

95

86

83

99

86

100

100

99

88

94

80

72

100

0.02

Recent Human H3N2 IVs

Recent Italian H1N2 strains

European H1N2 SIVs

European H3N2 SIVs

Port Chalmers 73-like

Italian H1N2 reassortant strains
