# Supplementary figures and images for: Different evolutionary trends of swine H1N2 influenza viruses in Italy compared to European viruses
Source: Vet Res. 2013 Dec 1;44(1):112. doi: 10.1186/1297-9716-44-112 (PMC4176092; doi:10.1186/1297-9716-44-112)

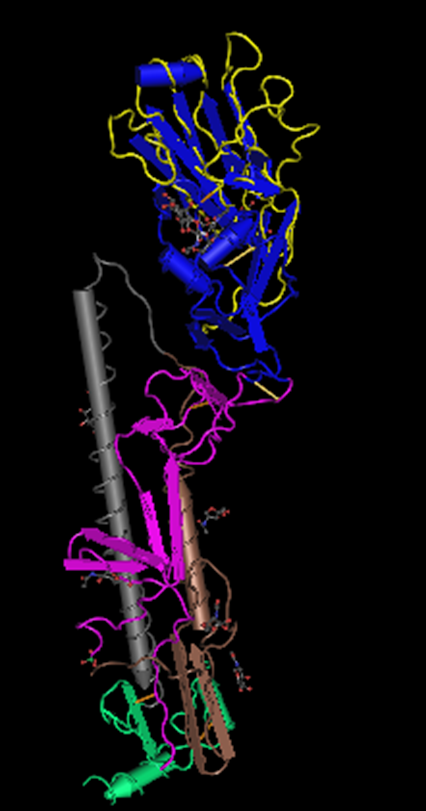


**R133-133A**

**a**

**Receptor binding site**


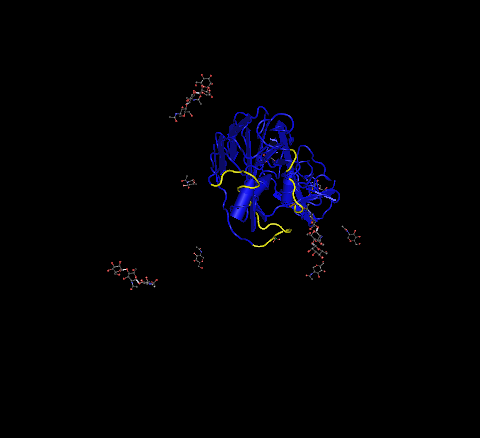


**b**

**220-loop R217-228**

**190-helix R190-195**

**130-loop R133-138**

**R133-133A**

Supplement: Additional file 3 — Predicted 3D-MMS of monomer of the H1 protein (a: lateral view and b: top view). Amino acid residues R133 and 133A, which are deleted in the recent Italian H1N2 strains, are shown in red. Numbering is expressed in H3 numbering. The receptor-binding subdomain, which is located in the globular part of the molecule, and the three secondary structure units making up the site are shown in yellow [35,36]. [file 1297-9716-44-112-S3.docx]
